# Influence of circadian preference, sleep inertia and their interaction on marathon completion time: A retrospective, cross‐sectional investigation of a large mass‐participation city marathon

**DOI:** 10.1111/jsr.14375

**Published:** 2024-10-19

**Authors:** Matthew K. P. Gratton, Jonathan Charest, James Lickel, Amy M. Bender, Penny Werthner, Charles R. Pedlar, Courtney Kipps, Doug Lawson, Charles H. Samuels, Jesse Cook

**Affiliations:** ^1^ Division of Medical Informatics, Department of Internal Medicine University of Kansas Medical Center Kansas City Kansas USA; ^2^ Social and Behavioral Sciences, Psychology University of Kansas Lawrence Kansas USA; ^3^ Université Laval, École de Psychologie Québec City Québec Canada; ^4^ Idorsia Pharmaceuticals Montréal Quebec Canada; ^5^ Department of Psychiatry University of Wisconsin‐Madison Madison Wisconsin USA; ^6^ William S. Middleton Mémorial Veterans Hospital Madison Wisconsin USA; ^7^ Faculty of Kinesiology University of Calgary Calgary Alberta Canada; ^8^ Absolute Rest Austin Texas USA; ^9^ Faculty of Sport, Allied Health, and Performance Science St Mary's University London UK; ^10^ Institute of Sport, Exercise and Health London UK; ^11^ Centre for Sleep and Human Performance Calgary Alberta Canada; ^12^ Faculty of Medicine University of Calgary Calgary Alberta Canada; ^13^ Department of Psychology University of Wisconsin‐Madison Madison Wisconsin USA

**Keywords:** circadian preference, endurance athletes, marathon, running performance, sleep inertia

## Abstract

Burgeoning interest in marathons necessitates an understanding of performance determinants. Research has highlighted the importance of diet, training and sleep, yet relations of circadian preference and sleep inertia with marathon performance remain largely unexplored. Because marathons generally start early‐to‐mid morning, these characteristics may have relevant impact. This study investigates relationships of circadian preference, sleep inertia and their interaction with marathon completion time. Consenting participants in a 2016 large mass‐participation city marathon completed self‐report questionnaires capturing circadian preference and sleep inertia, along with demographics and other characteristics. Circadian preference and sleep inertia were described across subgroups. Analyses examined the associations and interactions of circadian preference and sleep inertia with marathon completion times, with adjusted analyses accounting for age, sex and sleep health. Participants were marathon finishers (*n* = 936; 64.5% male; 66.3% young‐adults), with a majority reporting morningness tendencies (60.8%). Results supported a linear association between increasing eveningness preference with slower marathon times (*p* = 0.003; *p*_adjusted_ = 0.002), while some support was provided for a linear relationship between greater sleep inertia and slower marathon times (*p* = 0.04; *p*_adjusted_ = 0.07). A significant interaction was observed (*p* = 0.02; *p*_adjusted_ = 0.01), with the directionality suggesting that the circadian preference relationship weakened when sleep inertia severity increased, and vice‐versa. Our results suggest deleterious associations of increasing eveningness preference and greater sleep inertia with marathon completion time. These features may aid identifying marathoners who could be at a disadvantage, while also serving as modifiable targets for personalized training regimens preceding competition.

## 1 INTRODUCTION

Running as an exercise, hobby and sport is increasing across the population, with this translating into an uptick in population‐wide participation in marathons (Reusser et al., 2021). The increase in marathon participation has also coincided with more scientific efforts to identify factors that influence performance, which may be useful for guiding training regimes to enhance race‐day performance for marathoners of all skills and capabilities. Previous research has shown that endurance running performance may be influenced by individual (e.g. age and sex; Besson et al., 2022; Cook et al., 2023; Nikolaidis et al., 2019), lifestyle (e.g. diet and sleep; Burke et al., 2019; Costa et al., 2019; Furber et al., 2021; Lopes et al., 2023) and training characteristics (Carrasco‐Poyatos et al., 2022; Haugen et al., 2022; Prieto‐González & Sedlacek, 2022). However, this area of research is still relatively understudied, limiting the progression of efficacious, individualizable training strategies and protocols.

The circadian rhythm, or “biological clock”, is a key component of physiology that plays a central role in regulating behaviour and functioning, including the sleep and wake cycle (Dibner et al., 2010; Neves et al., 2022). An abundance of research exists showcasing the influence of the circadian rhythm on athletic performance across a multitude of sports (Ayala et al., 2021; Nobari et al., 2023). Humans differ in the timing of their circadian rhythms, with individuals falling along a circadian spectrum spanning morningness to eveningness extremes (Putilov, 2017). Circadian preference is a term used to capture one's subjective tendency towards a particular circadian type that often reflects a sleep–wake schedule. Research has shown that the timing of peak performance in athletes is dependent upon one's circadian preference (Facer‐Childs & Brandstaetter, 2015). Given that marathons generally begin in early‐ to mid‐morning, an individual's circadian preference may be a uniquely important factor for race‐day performance.

Sleep inertia is a term used to capture the period of physical, cognitive and psychological impairment that occurs immediately after awakening from sleep (Hilditch & McHill, 2019). Sleep inertia is a normal experience, yet humans will differ in the duration and intensity of sleep inertia based on situational and trait‐level factors (Hilditch, Pradhan, Costedoat, Bathurst, Glaros, Gregory, & Flynn‐Evans, 2023; Lundholm et al., 2021; Trotti, 2017). With the relatively early start times of marathons, participants with more prolonged, severe sleep inertia may be at a performance disadvantage. Fortunately, research suggests that sleep inertia severity may be modifiable through countermeasures (Hilditch et al., 2022; Hilditch, Pradhan, Costedoat, Bathurst, Glaros, Gregory, Shattuck, & Flynn‐Evans, 2023). However, to our knowledge, no previous investigation has evaluated the relationship between sleep inertia features and marathon or endurance running performance.

The present study was designed to advance the literature by evaluating the associations of circadian preference, sleep inertia and their interaction with marathon performance (i.e. marathon completion time). The first aim of this study was to describe circadian preference and sleep inertia in this sample of marathon participants, as well as within subgroups based on available demographic and lifestyle characteristics. Additionally, we aimed to assess the relationships of circadian preference and sleep inertia with marathon completion time, with and without controlling for relevant, available individual characteristics. We approached this study with the hypothesis that both greater degrees of eveningness and more severe sleep inertia severity would associate with slower marathon completion time. We also hypothesized an interactive relationship between circadian preference and sleep inertia, whereby greater sleep inertia would strengthen the relationship of circadian preference with marathon completion time (and vice‐versa).

## 2 METHODS

### 2.1 Data collection, ethical approval and oversight, analytic sample, and race details

Participants in a 2016 large mass‐participation city marathon were approached directly in‐person during the event registration process over a 4‐day period. Interested marathoners were provided detailed information about the investigation from a study team member. They were informed that completion of the measures associated with the study indicated consent, while also being informed that they had the opportunity to email the researchers to obtain their results on the measures. All study procedures were approved by the St Mary's University Ethics Committee (Twickenham, London, UK).

The initial dataset included data from consenting participants who completed the marathon, which included responses from 951 marathoners. This dataset was reduced to construct the analytic dataset. After evaluating the distribution of marathoners across age, initially provided as 18–39 years, 40–44 years, 45–49 years, 50–54 years, 55–59 years, 60–64 years, 65–69 years, and 70 years and over (70+), we determined it best to create an age variable with fewer levels. We explored a three categorical variable approach (young [18–39 years], middle‐aged [40–64 years] and older adults [65+ years]). Yet, older adults were scarce in the sample (*n* = 8; 0.84% of the total sample). Given this, we decided to omit this small percentage of marathoners from the analytic sample. Another seven participants were removed due to absence of responses on questions used to characterize the focal variables of interest.

Marathoners completed a 26.2‐mile (~42.2 km) course (TCS London Marathon, 2023). In terms of marathon start time, the mass start time was 10:00 hours BST, which also included the elite men division as well as the British Athletics and England Athletics Championships for Men and Women. The elite women division start time was 09:15 hours BST (Marsden, 2016).

### 2.2 Collected measures and variable preparation

#### 2.2.1 Collected measures

All participants completed the Athlete Sleep Screening Questionnaire (ASSQ), along with a general, brief survey. The ASSQ is a valid and reliable measure designed specifically for assessing sleep health in athletic populations (Bender et al., 2018), while also capturing other information related to circadian preference, sleep inertia and relevant lifestyle information. Full details on the ASSQ can be found elsewhere (Bender et al., 2018; Samuels et al., 2016).

#### 2.2.2 Focal variables: Circadian preference, sleep inertia and marathon completion time

Circadian preference and sleep inertia were derived from single items in the ASSQ. Circadian preference was derived from a question asking whether the participant considered themselves to be a morning or evening person, with four potential responses: Definitely a morning type (Morning); More a morning type than evening type (Morning > Evening); More an evening type than a morning type (Evening > Morning); Definitely an evening type (Evening). This variable was prepared as a categorical variable with the four response levels as well as a continuous variable (1–4), with higher values reflecting greater degrees of eveningness. Sleep inertia was characterized from a question asking about the participant's level of alertness during the first half‐hour after awakening, with four potential responses: Not at all alert; Slightly alert; Fairly alert; and Very alert. This variable was prepared as a categorical variable with the four response levels as well as a continuous variable (1–4), with higher values reflecting greater degrees of sleep inertia (less alertness). Marathon completion time was collected for all participants, and formatted in minutes across analyses.

#### 2.2.3 Supplementary variables: Sex, age, runner performance based on expectation, and sleep difficulty score (SDS), as well as alcohol, caffeine, electronic device and sleep tracker use

Participants provided a self‐report of whether they were male or female on the general brief survey. Although not entirely clear whether this reflects gender or sex, the research team deemed it most likely to represent sex, with this variable dichotomized for analyses (female versus male). We previously described (see Section 2.1) the collapsing of the six age groups included in the analytic dataset into a dichotomized age variable (young versus middle‐aged adults). Young adults were defined as the 18–39 years old age group, while 40–64 years old defined the middle‐aged group. A dichotomized variable was created to represent race day performance based on expectation. We compared a participant's marathon completion time against their “Good For Age (GFA)” qualifying threshold to create groups of those who completed the marathon faster than their GFA (GFA‐Faster) versus those who were slower than their GFA (GFA‐Slower; TCS London Marathon, 2024). The GFA was determined based on participant's age division for the race (e.g. 18–39 years, 40–44 years, 45–49 years, etc.) and not the dichotomized age variable used in analyses (e.g. young versus middle‐aged adults).

The ASSQ provided assessment of participants' SDS, use of a sleep tracking device, weekly alcohol, daily caffeine use, and weekly electronic device use within 1 hr of bedtime. The SDS is computed from the summation of five items related to sleep duration, satisfaction/dissatisfaction with sleep quality, sleep‐onset latency, sleep maintenance, and sleep medication use. Four severity thresholds have been established for the SDS: none (0–4); mild (5–7); moderate (8–10); and severe (11–17). Research has also implicated a SDS ≥ 8 as a clinically significant threshold for athletes, which was used in this investigation to create a dichotomized version of SDS (below versus above threshold; Bender et al., 2018). Dichotomized versions of sleep tracker user (yes versus no), weekly alcohol use (low/no versus higher), daily caffeine use (low/no versus higher), and weekly electronic device use within 1 hr of bedtime (low/no versus higher) were also created for subgroup analyses. For alcohol use, the higher characterization comprised of participants reporting consuming eight or more drinks per week. The higher characterization for daily caffeine use captured the participants who provided responses reflecting three or more caffeinated drinks per day. The higher characterization for weekly electronic device use within 1 hr of bedtime captured the participants who provided responses reflecting use on a majority of nights (four or more per week).

### 2.3 Statistical analyses

Descriptive analyses were performed to characterize the sample across focal and supplementary variables, as well as marathon completion time.

In analyses that evaluated circadian preference and sleep inertia across subgroups, linear regression was employed. Circadian preference and sleep inertia served as outcome variables, separately, across these analyses, with continuous variable versions utilized. The dichotomized versions of supplementary variables were utilized as predictors to assess circadian preference and sleep inertia across sex, age, runner performance based on expectation, and SDS, as well as alcohol (weekly), caffeine (daily), electronic device use within 1 hr of bedtime (weekly), and sleep tracker use. Means and standard deviations for the subgroups are provided, along with the regression coefficient (*β*), standard error (SE), lower‐ and upper‐limit from the 95% confidence interval, *p*‐value, and partial eta squared ($\eta_{p}^{2}$) for each regression. $\eta_{p}^{2}$ is a commonly employed measure of effect size that reflects the proportion of variance explained by an independent variable, ranging from 0 to 1 (Richardson, 2011). When performing univariate analyses, $\eta_{p}^{2}$ is equal to the coefficient of determination (*R* ^2^) and is used in multivariable models to reflect the unique explanatory value for an independent variable while accounting for the effects of other independent variables in the model. Multiplying $\eta_{p}^{2}$ by 100 results in the percentage of explained variance for a specific independent variable.

The focal analyses evaluated the relationships of circadian preference and sleep inertia with marathon completion time. Analysis of variance (ANOVA) was utilized to assess for significant variance across the groups in circadian preference and sleep inertia across marathon completion time, respectively. Post‐hoc pairwise comparisons were performed, with additional analyses correcting for multiple comparisons using Tukey HSD, which is a common approach for controlling the overall Type I error rate (Williams et al., 1999). Regression analysis was also employed to assess for linear relationships of circadian preference and sleep inertia with marathon completion time. Unadjusted regressions analyses were performed that just included circadian preference or sleep inertia as the predictor with marathon completion time as the outcome, as well as adjusted analyses that included the covariates of age, sex and SDS (modelled continuously as a covariate). Lastly, we explored an interactive relationship of circadian preference and sleep inertia on marathon completion time by fitting a model that regressed marathon completion time on circadian preference, sleep inertia and their interaction. An adjusted version of the model was also performed that accounted for age, sex and SDS.

Data preparation and analyses for this project were performed in RStudio v4.3.0 (RStudio, Boston, MA, USA).

## 3 RESULTS

### 3.1 Sample characteristics

The analytic sample (*n* = 936) was predominantly male (64.5%), young adults (66.3%) and completed the marathon at a slower pace than their GFA (78.4%). The sample reflected 2.39% of all race starters and 2.41% of the total race finishers. Morningness tendencies (Definitely a morning type, or More a morning type than evening type) were reported by the majority of the sample (60.8%; Table 1). Sleep inertia severity problems were low across the sample, with the majority (64.3%) reporting either being Very or Fairly alert within the first half‐hour after awakening; and 23.4% of the sample reported sleep difficulty severities above the ASSQ's established clinically significant threshold. Sleep tracker use was uncommon across the sample, with 88.4% of the participants reporting not using a sleep tracker. Electronic device use within 1 hr of bedtime was common, with 58.2% of the sample reporting this behaviour every day. The majority of the sample reported low daily caffeine use (≤ 2 drinks; 51.7%), with a notable number of absent responses for this variable (12.7%). Higher alcohol use across the week (≥ 8 drinks) was uncommon (12.9% of sample). Average marathon completion time was 250 min (Table 1).

**TABLE 1:** Descriptive statistics across analytic sample.

Characteristic	Category	%
Sample size (*N*)	936	
Gender	Female	35.50%
	Male	64.50%
Marathon completion time		250 ± 52.9 min
Age group percentage (marathon designation)	18–39 years	66.30%
	40–44 years	15.60%
	45–49 years	10.10%
	50–54 years	5.45%
	55–59 years	1.50%
	60–64 years	0.96%
Age group percentage (statistical analyses)	Young adults (18–39 years)	66.30%
	Middle‐aged adults (40–64 years)	33.70%
Runner performance (GFA qualifying threshold)	Faster than GFA	21.60%
	Slower than GFA	78.40%
Circadian preference	Definitely morning	33.80%
	Morning > Evening	27.00%
	Evening > Morning	25.90%
	Definitely evening	13.40%
Sleep inertia	Very alert	16.90%
	Fairly alert	47.40%
	Slightly alert	27.50%
	Not at all alert	8.23%
Global SDS	None	35.70%
	Mild	40.90%
	Moderate	18.20%
	Severe	5.24%
Bedtime electronic device use (per week)	1–3 ×	15.80%
	4–6 ×	19.90%
	Everyday	58.20%
	Not at all	5.24%
	No response	0.96%
Sleep tracker user	Yes	11.50%
	No	88.40%
	No response	0.10%
Caffeine use (per day)	< 1	18.90%
	2‐Jan	32.80%
	3	22.20%
	4	13.40%
	5+	0.00%
	No response	12.70%
Alcohol use (per week)	Does not drink	14.40%
	< 2 drinks	24.30%
	2–4 drinks	27.40%
	5–7 drinks	20.40%
	8–14 drinks	9.83%
	> 14 drinks	3.10%
	No response	0.64%

Sample characteristics for the project. Available individual characteristics include self‐identified, binary gender (male or female), age group during the London Marathon, and runner performance on race day based on completion time relation to “Good for Age (GFA)” qualifying standards (GFA‐Faster or GFA‐Slower). Circadian preference was characterized based on responder's choice of Definitely morning, More morning than evening (Morning > Evening), More evening than morning (Evening > Morning), and Definitely evening. Sleep inertia was characterized based on responses to an item inquiring about alertness within the first half‐hour of awakening, with options including: Very alert; Fairly alert; Slightly alert; and Not at all alert. SDS severity characterization is provided based on previously determined thresholds. Other, available characteristics included nightly electronic use within 1 hr of bedtime, whether or not the marathoner was a sleep tracker user, daily caffeine use, and weekly alcohol use. Values are presented as proportions across the entire sample, except for sample size (count) and marathon completion time (mean ± standard deviation).

GFA, Good For Age; SDS, sleep difficulty score.

### 3.2 Circadian preference and sleep inertia across marathon subgroups

Table 2 presents the analyses evaluating differences in circadian preference and sleep inertia across subgroups. Subgroups were dichotomized as predictors for these analyses, and included sex, age, runner performance based on expectation, and SDS, as well as alcohol (weekly), caffeine (daily), electronic device within 1 hr of bedtime (weekly), and sleep tracker use.

**TABLE 2:** Circadian preference and sleep inertia across subgroups.

Variable	CP/SI	Group	Mean ± SD	*β*	SE	95% CI	*p*‐Value	$\eta_{p}^{2}$
Age group	CP	Young adults	2.27 ± 1.04	0.23	0.07	0.09, 0.37	0.001	0.011
		Middle‐aged adults	2.04 ± 1.04					
	SI	Young adults	2.33 ± 0.83	0.18	0.06	0.07, 0.30	0.002	0.011
		Middle‐aged adults	2.15 ± 0.83					
Gender	CP	Female	2.14 ± 1.05	−0.07	0.07	−0.07, 0.21	0.31	0.001
		Male	2.21 ± 1.05					
	SI	Female	2.27 ± 0.87	0	0.06	−0.11, 0.12	0.95	0
		Male	2.27 ± 0.82					
SDS threshold	CP	Below threshold	2.18 ± 1.03	−0.05	0.08	−0.21, 0.11	0.52	0.001
		Above threshold	2.23 ± 1.09					
	SI	Below threshold	2.25 ± 0.84	−0.11	0.07	−0.23, 0.02	0.1	0.003
		Above threshold	2.35 ± 0.84					
GFA status	CP	Slower	2.25 ± 1.05	0.28	0.08	0.12, 0.44	< 0.001	0.012
		Faster	1.97 ± 1.01					
	SI	Slower	2.29 ± 0.82	0.09	0.07	−0.04, 0.22	0.17	0.002
		Faster	2.20 ± 0.88					
Caffeine use	CP	Low/No	2.20 ± 1.05	0.04	0.07	−0.11, 0.18	0.62	0
		Higher	2.17 ± 1.04					
	SI	Low/No	2.30 ± 0.84	0.09	0.06	−0.20, 0.02	0.11	0.003
		Higher	2.21 ± 0.82					
Alcohol use	CP	Low/No	2.16 ± 1.04	−0.2	0.1	−0.04, −0.00	0.05	0.004
		Higher	2.36 ± 1.11					
	SI	Low/No	2.26 ± 0.83	−0.09	0.06	−0.25, 0.07	0.28	0.001
		Higher	2.35 ± 0.85					
E‐device use	CP	Low/No	2.00 ± 1.06	−0.24	0.08	−0.40, −0.08	0.004	0.009
		Higher	2.24 ± 1.04					
	SI	Low/No	2.27 ± 0.84	0	0.07	−0.13, 0.13	0.95	0
		Higher	2.27 ± 0.84					
Sleep tracker use	CP	No	2.19 ± 1.05	0.05	0.11	−0.27, 0.26	0.61	0
		Yes	2.14 ± 1.00					
	SI	No	2.25 ± 0.82	−0.17	0.09	−0.00, 0.33	0.05	0.004
		Yes	2.42 ± 0.94					

Circadian preference (CP) and sleep inertia (SI) analyses across subgroups within the sample. For these analyses, CP was coded on a 1–4 scale, with increasing values representative of greater eveningness preference: (1) Definitely morning; (2) More morning than evening; (3) More evening than morning; (4) Definitely evening. SI was coded on a 1–4 scale, with increasing values representative of decreased alertness within the first hour of awakening: (1) Very alert; (2) Fairly alert; (3) Slightly alert; (4) Not at all alert. Dichotomized subgroups were established for age (young versus middle‐aged adults), sex (female versus male), clinically suggestive SDS threshold from the athlete sleep screening questionnaire (below versus above), marathon completion time relative to GFA qualifying time (faster versus slower), daily caffeine use (low/no versus higher), weekly alcohol use (low/no versus higher), weekly electronic device use (E‐device) in the hour preceding bedtime (low/no versus higher), and whether the participant was a sleep tracker user (yes versus no). Linear regression was utilized to evaluate subgroups across CP and SI outcomes, separately, with the regression coefficient (β), standard error (SE), lower‐ and upper‐limits from the 95% CI, *p*‐value and partial eta squared ($\eta_{p}^{2}$) presented. Group means ± standard deviation are also presented.

CI, confidence interval; GFA, Good For Age; SDS, sleep difficulty score.

Younger adults reported significantly greater eveningness tendencies (*β* = 0.23; *p* = 0.001), as well as greater sleep inertia severity (*β* = 0.18; *p* = 0.002), relative to middle‐aged adult marathoners. Marathoners who performed slower than their GFA qualifying threshold (GFA‐Slower) reported significantly greater eveningness (*β* = 0.28; *p* < 0.001) relative to GFA‐Faster. Those who reported low‐to‐no weekly alcohol use reported significantly greater morningness (*β* = −0.20; *p* = 0.05), relative to the higher weekly alcohol use marathoners. Significantly greater morniningness tendencies were also observed in marathoners who reported low‐to‐no frequency of electronic device use within 1 hr of bedtime (*β* = −0.24; *p* = 0.004), relative to those with higher electronic device use within 1 hr of bedtime. Marathoners who were not using a sleep tracking device reported less sleep inertia severity (*β* = −0.17; *p* = 0.05), relative to those using a sleep tracking device. No other statistically significant differences in circadian preference or sleep inertia across subgroups were observed.

### 3.3 Relationships between circadian preference, sleep inertia and their interaction with marathon performance

**FIGURE 1:**
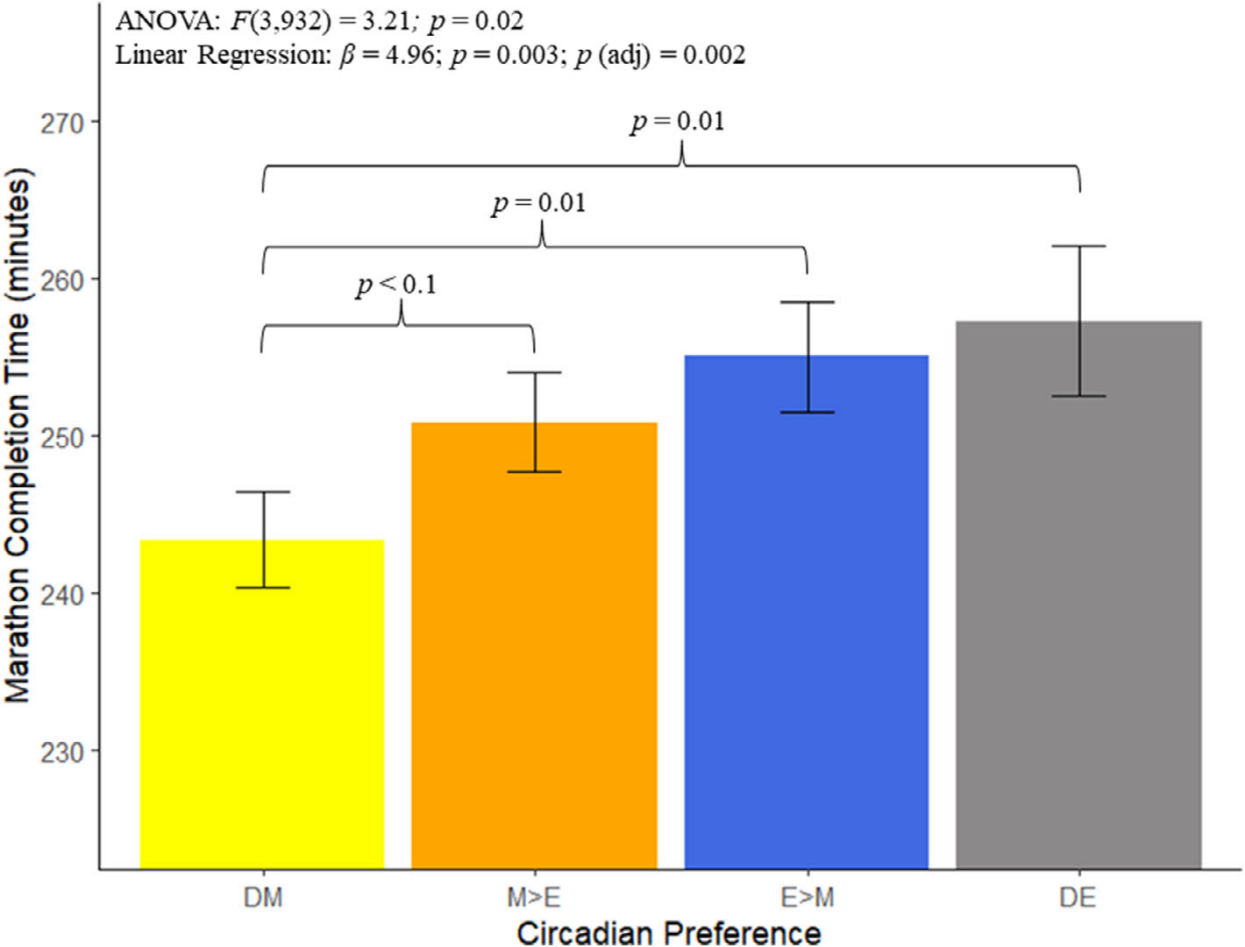
The main results from the analyses of circadian preference with marathon completion time (min). The average marathon completion time and standard error are plotted across the four levels of circadian preference: Definitely a morning type (DM); More a morning type than evening type (M > E); More an evening type than a morning type (E > M); and Definitely an evening type (DE). The *F* statistic and associated *p*‐value are presented from the ANOVA, along with the regression coefficient (*β*) and associated *p*‐value from the unadjusted linear regressions, as well as the *p*‐value from the adjusted regressions. Significant (*p* < 0.05) and trend level significant (*p* < 0.1) relationships from pairwise comparisons are also noted.

**FIGURE 2:**
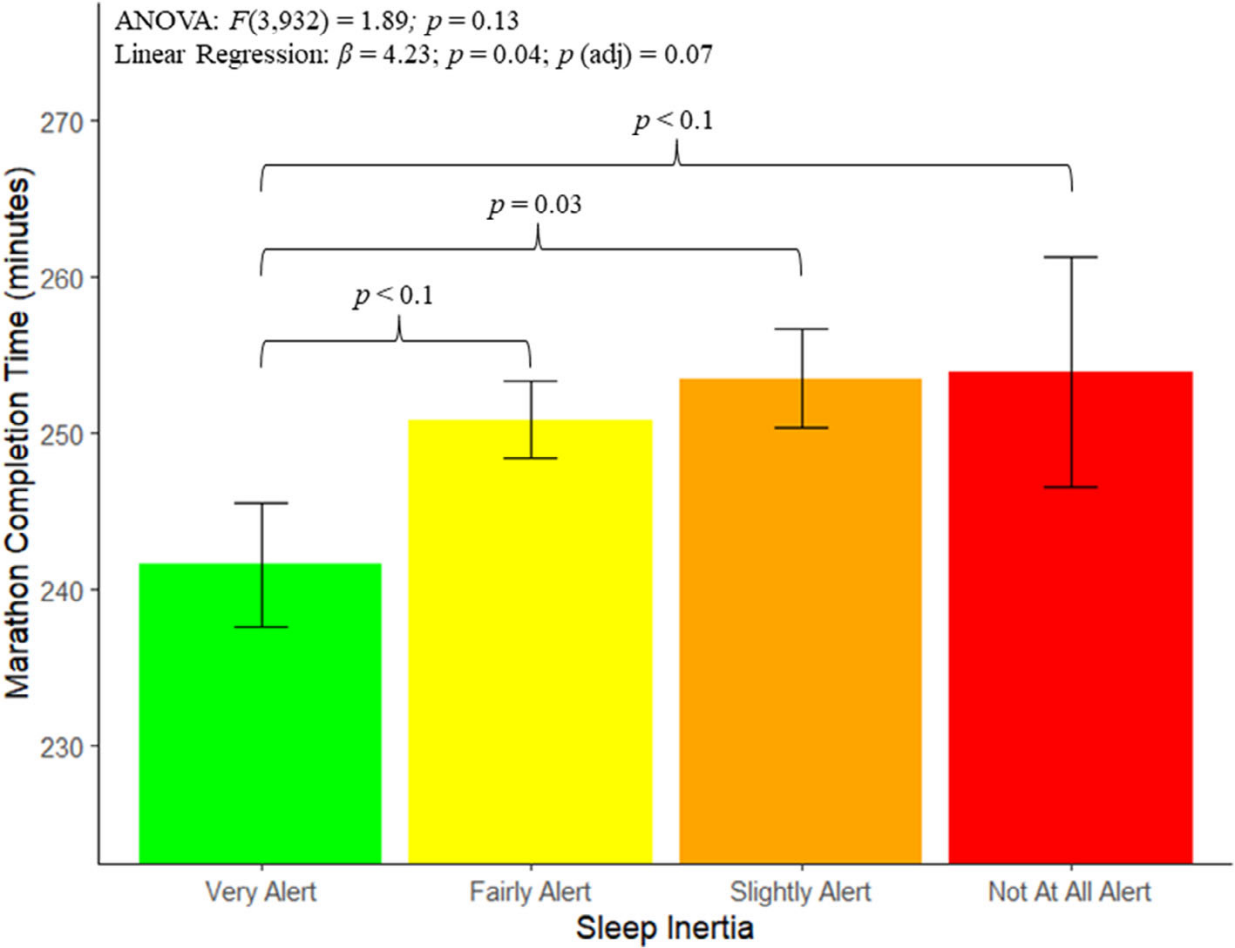
The main results from the analyses of circadian preference and sleep inertia with marathon completion time (min). The average marathon completion time and standard error are plotted across the four levels of sleep inertia: Very alert; Fairly alert; Slightly alert; and Not at all alert. The *F* statistic and associated *p‐*value are presented from the ANOVA, along with the regression coefficient (*β*) and associated *p*‐value from the unadjusted linear regressions, as well as the *p*‐value from the adjusted regressions. Significant (*p* < 0.05) and trend level significant (*p* < 0.01) relationships from pairwise comparisons are also noted.

The relationships between circadian preference, sleep inertia and their interaction with marathon performance (captured by marathon completion time) were the focal analyses. Figure 1 presents the relationship between circadian preference and marathon completion time, while Figure 2 presents the relationship between sleep inertia and marathon completion time. Table 3 presents the average completion time across circadian preference groups, while Table 4 presents the results from the pairwise comparisons between circadian preference groups. Table 5 presents the average completion time across sleep inertia groups, while Table 6 presents the results from the pairwise comparisons between sleep inertia groups.

**TABLE 3:** Marathon completion time, runner performance, gender and age across circadian preference.

Characteristic	Definitely morning	Morning > evening	Evening > morning	Definitely evening
Sample size (*N*)	316	253	242	125
Marathon completion time (min)	244 ± 53.6	251 ± 49.9	255 ± 53.9	257 ± 53.3
Runner performance (%)				
Faster than GFA	27.2	21.7	17.4	15.2
Slower than GFA	72.8	78.3	82.6	84.8
Gender (%)				
Male	63.0	62.1	69.4	64.0
Female	37.0	37.9	30.6	36.0
Age group—marathon divisions (%)				
18–39 years	58.5	70.4	68.6	73.6
40–44 years	17.1	12.7	16.9	15.2
45–49 years	14.6	9.88	7.44	4.8
50–54 years	6.33	5.93	4.55	4.0
55–59 years	1.9	0.79	1.65	1.6
60–64 years	1.58	0.4	0.83	0.8
Age group—statistical analyses (%)				
Young adults (18–39 years)	58.5	70.4	68.6	73.6
Middle‐aged adults (40–64 years)	41.5	29.6	31.4	26.4

Sample size, marathon completion time, runner performance based on GFA qualifying time, gender, age group based on marathon divisions, and age group based on the dichotomized age variable used for statistical analyses across the circadian preference groups: Definitely morning, Morning > Evening, Evening > Morning, and Definitely evening. Sample size is presented as counts (*n*), with marathon completion time provided as means ± standard deviation, and all other characteristics described as percentage within a circadian preference group.

GFA, Good For Age.

**TABLE 4:** Pairwise comparisons of circadian preference groups: Definitely morning, Morning > Evening, Evening > Morning, and Definitely evening.

Comparison	MD (min)	SE	*p*	*p* (corrected)
Definitely morning versus Morning > Evening	7.46	4.41	0.09	0.34
Definitely morning versus Evening > Morning	11.6	4.5	0.01	0.05
Definitely morning versus Definitely evening	13.9	5.52	0.01	0.06
Morning > Evening versus Evening > Morning	4.18	4.74	0.38	0.81
Morning > Evening versus Definitely evening	6.44	5.71	0.26	0.68
Evening > Morning versus Definitely evening	2.26	5.8	0.7	0.98

Mean difference, standard error (SE), *p*‐value and Tukey HSD corrected *p*‐value are presented. Pairwise comparisons between circadian preference groups for marathon completion time. Mean difference (MD), standard error (SE), *p*‐value and a Tukey HSD corrected *p*‐value [*p* (corrected)] are presented for each comparison. MD is presented in minutes, with the value reflecting the second group minus the first group (e.g. Morning > Evening—Definitely morning = 7.46).

**TABLE 5:** Age, gender, marathon completion time and runner performance across sleep inertia.

Characteristic	Very alert	Fairly alert	Slightly alert	Not at all alert
Sample size (*N*)	158	444	257	77
Marathon completion time (min)	242 ± 50.3	251 ± 52.3	254 ± 51.2	254 ± 64.4
Runner performance (%)				
Faster than GFA	27.2	21.3	17.5	24.7
Slower than GFA	72.8	78.6	82.5	75.3
Gender (%)				
Male	61.4	65.3	67.3	57.1
Female	38.6	34.7	32.7	42.9
Age group—marathon divisions (%)				
18–39 years	58.2	64.4	73.2	71.4
40–44 years	20.9	13.5	16.0	15.6
45–49 years	10.1	13.1	6.62	5.2
50–54 years	6.33	6.53	3.11	5.2
55–59 years	1.9	1.58	1.17	1.3
60–64 years	2.53	0.9	0	1.3
Age group—statistical analyses (%)				
Young adults (18–39 years)	58.2	64.4	73.2	71.4
Middle‐aged adults (40–64 years)	41.8	35.6	26.9	28.6

Sample size, marathon completion time, runner performance based on GFA qualifying time, gender, age group based on marathon divisions, and age group based on the dichotomized age variable used for statistical analyses across the sleep inertia groups: Very alert; Fairly alert; Slightly alert; and Not at all alert. Sample size is presented as counts (*n*), with marathon completion time provided as means ± standard deviation, and all other characteristics described as percentage within a circadian preference group.

GFA, Good For Age.

**TABLE 6:** Pairwise comparisons of sleep inertia groups: Very alert; Fairly alert; Slightly alert; Not at all alert.

Comparison	MD (min)	SE	*p*	*p* (corrected)
Very alert versus Fairly alert	9.26	4.89	0.06	0.23
Very alert versus Slightly alert	11.9	5.33	0.03	0.12
Very alert versus Not at all alert	12.4	7.33	0.09	0.33
Fairly alert versus Slightly alert	2.65	4.24	0.52	0.92
Fairly alert versus Not at all alert	3.09	6.52	0.47	0.97
Slightly alert versus Not at all alert	0.44	6.86	0.95	1.00

Mean difference, standard error (SE), *p*‐value, and Tukey HSD corrected *p*‐value are presented. Pairwise comparisons between sleep inertia groups for marathon completion time. Mean difference (MD), standard error (SE), *p*‐value and a Tukey HSD corrected *p*‐value [*p* (corrected)] are presented for each comparison. MD is presented in minutes, with the value reflecting the second group minus the first group (e.g. Fairly alert—Very alert = 9.26).

The ANOVA results evidenced significant variance in marathon completion time across circadian preference (*F*_3,932_ = 3.21; *p* = 0.02). Follow‐up pairwise comparisons evidenced statistically significant differences in the comparisons between Definitely morning and Evening > Morning preference groups (mean difference = 11.6 min; *p* = 0.01), and Definitely morning and Definitely evening preference groups (mean difference = 13.9 min; *p* = 0.01). The relationships suggested that morningness associated with faster marathon completion time, relative to eveningness groups. However, after correcting for multiple comparisons, statistical significance was only observed for the Definitely morning and Evening > Morning relationship (*p* = 0.05), with trend level significance observed for the Definitely morning and Definitely evening relationship (*p* = 0.06). When assessed linearly, greater degrees of eveningness significantly associated with longer marathon completion time (*β* = 4.96; SE = 1.64; 95% confidence interval [CI] = [1.73, 8.19]; *p* = 0.003; $\eta_{p}^{2}$ = 0.010), with significance also observed in the adjusted model that accounted for marathon runner age, sex and SDS severity (*p*_adj_ = 0.002; $\eta_{p}^{2}$_adj_ = 0.011).

The ANOVA results did not evidence significant variance in marathon completion time across sleep inertia severities. When assessed linearly, greater degrees of sleep inertia severity significantly associated with longer marathon completion time (*β* = 4.23; SE = 2.06; 95% CI = [0.18, 8.27]; *p* = 0.04; $\eta_{p}^{2}$ = 0.005), yet only trend level significance was observed in the adjusted model that accounted for marathon runner age, sex and SDS severity (*p*_adj_ = 0.07; $\eta_{p}^{2}$_adj_ = 0.003).

To test for an interactive relationship between circadian preference and sleep inertia on marathon completion time, a model was fitted that regressed marathon completion time on circadian preference, sleep inertia and an interaction term between the two continuous variables. The results evidenced a statistically significant interaction (*β* = −4.37, *p* = 0.02), with significance also observed in an adjusted model that accounted for marathon runner age, sex and SDS severity (*p*_adj_ = 0.01). The directionality of the coefficient suggests that as the degree of sleep inertia severity increases, the effect of circadian preference on marathon completion decreases. Similarly, as circadian preference shifts towards eveningness, the effect of sleep inertia severity on marathon completion time decreases. Figures S1 and S2 visually present the interactive relationship between circadian preference and sleep inertia on marathon completion time.

## 4 DISCUSSION

Population‐wide interest and participation in marathons has increased. Marathon popularity has coincided with scientific pursuit of individual, lifestyle and training factors that influence race‐day performance. This is useful for guiding the development of individualized training regimes. However, relations of circadian preference and sleep inertia with marathon performance are understudied, despite the fact that these characteristics may have unique import given typical marathon start times. This investigation was designed to advance this gap in knowledge by evaluating the associations of circadian preference, sleep inertia and their interaction with marathon completion time, using data collected from participants in a 2016 large mass‐participation city marathon. Our results provided consistently strong evidence for the association between greater degrees of eveningness preference and longer marathon completion times (worse performance). Additionally, we observed some statistical support for a relationship between greater sleep inertia severity and longer marathon completion times. A significant, positive interaction between circadian preference and sleep inertia on marathon completion time was also observed, yet the directionality of this interaction was not in alignment with our a priori hypothesis as it suggested that the linear relationship between increasing degrees of circadian preference and longer marathon completion time weakened with greater degrees of sleep inertia, and vice‐versa.

The combination of results from descriptive, regression and ANOVA analyses highlighted the benefit of being *Definitely morning*, as well as the association of greater eveningness preference with worse marathon completion times for a marathon beginning at a traditional early‐ to mid‐morning start time. These results match our hypotheses and may merely reflect the benefits of alignment in circadian biology with competition timing. Yet, there are additional layers to this relationship that warrant attention. Other psychosocial factors and training dynamics may be relevant to understanding the potentially deleterious effects of eveningness. For example, our results also showed that greater eveningness associated with increased weekly alcohol use and greater frequency of electronic device use within 1 hr of bedtime, which are behavioural tendencies that are unlikely to aid training, recovery and race‐day performance. These behaviours may serve as targetable interventions for marathoners of eveningness circadian type that could enhance marathon performance. Additionally, future research aimed at evaluating the effects of modifying circadian preference and underlying circadian biology preceding race‐day competition through circadian‐based strategies (e.g. chronotherapy) is warranted. Advancing this line of research is critical to clarify the key elements and subsequent strategies for individualized training regimes that account for differences in circadian type.

Although we did not observe consistently significant results across regression and ANOVA analyses, the general pattern and significant, positive association in the unadjusted regression analyses provide some evidence supporting our hypothesis that greater sleep inertia severity would associate with worse marathon performance. Reducing sleep inertia through reactive countermeasures, such as light, sound and/or temperature, upon awakening may be useful strategies for enhancing early morning training and race‐day performance (Hilditch et al., 2016). However, it is important to note that the study of sleep inertia and potential therapeutic strategies is still in its relative infancy, with further progression in this area of research necessary to establish implementable strategies and protocols. It is also critical to note that we relied on a single item from the ASSQ that specifically focuses on the alertness/vigilance component of sleep inertia, while sleep inertia also is a physical and psychological experience. Future research should strive to utilize more comprehensive subjective measures of sleep inertia, such as the Sleep Inertia Questionnaire (Kanady & Harvey, 2015), as well as objective measures of sleep inertia features, such as the Psychomotor Vigilance Task (Evangelista et al., 2022), when possible, to enhance the understanding of the relationship between sleep inertia severity and marathon runner performance.

Although we approached this investigation expecting a significant interaction between circadian preference and sleep inertia on marathon completion time, the directionality of the observed, significant interaction from the results was surprising and antithetical to our expectations. We hypothesized that greater degrees of sleep inertia would augment a deleterious relationship between increasing eveningness preference and longer marathon completion time, and vice‐versa. However, the negative regression coefficient (*β* = −4.37) from the interaction analysis suggests that the deleterious relationship between increasing eveningness preference and longer marathon completion time weakens with greater sleep inertia severity. This finding is paradoxical and difficult to explain. Further evaluation of the relationship visually showed that the directionality of the interaction relationship is largely driven by the most severe sleep inertia phenotype (“Not at all alert”), whereby faster marathon completion times were observed in eveningness groups relative to the morningness groups for this sleep inertia subgroup. Although one can only speculate on the origin of this unforeseen relationship, perhaps this reflects differences in other unmeasured characteristics, such as a psychological trait related to grit or resilience. This may also be reflecting differences in training dynamics, such as volume and/or timing, that may have influenced greater sleep inertia while also resulting in better quality training. Future research is necessary to better understand the interrelation of circadian preference and sleep inertia with marathon completion time, as well as individual factors and traits that may moderate and mediate these relationships.

While our study provides novel insights, it is not without limitations. Our primary outcome of completion time served as a crude measure of marathon performance as it is possible that not all marathoners were trying to perform to their best; however, future studies should improve on this design, and could include exercise physiology variables such as velocity at a fixed blood lactate level, or critical speed data derived from training data (Smyth & Muniz‐Pumares, 2020). The cross‐sectional nature of our study design limits causal inferences. Additionally, the reliance on self‐reported measures for circadian preference and sleep inertia might introduce bias or inaccuracies. Furthermore, circadian preference may not directly map onto biological chronotype, which limits the ability to fully clarify the degree to which circadian biology is contributing to differences in marathon completion time. Moreover, the ASSQ item utilized to capture sleep inertia only focused on the alertness/vigilance feature of sleep inertia, which limited our ability to capture sleep inertia as it also is a physical and psychological experience. Sleep inertia characterization was also limited by the reliance on a single collection point, with variability in the collection time across participants. Because sleep inertia severity is likely to vary on different days, while also being influenced by a myriad of factors (e.g. time of collection; previous night sleep), future research should leverage longitudinal collection in a standardized manner to obtain a more accurate depiction of sleep inertia. We also did not have information on the specific start time of the marathon for each participant. Although it is very likely that most participants (and potentially all) began at the 10:00 hours start, it is possible that some women in the analytic sample competed in the elite division, which would lead to slight differences in start time (09:15 hours) that could have influenced the results. Lastly, while our sample was relatively large and diverse in terms of marathon completion times and demographics, it strictly focused on marathoners from a single event in 2016 and may not fully represent the global marathon‐running community. Future studies should strive to enhance the scope of captured demographic and lifestyle characteristics of the marathon participants.

## 5 CONCLUSION

To our knowledge, this is the first investigation to assess the interrelations among circadian preference, sleep inertia and marathon completion time. The relatively large sample size (*n* = 936) of marathoners is a notable strength of this investigation. Morningness tendencies were common within the sample, with 60.8% of participants reporting a circadian preference of either Definitely morning (33.8%) or Morning > Evening (27.0%). The majority of the sample reported low‐to‐moderate levels of sleep inertia, 74.9% reporting feeling Fairly alert (47.4%) or Slightly alert (27.5%) within the first half‐hour after awakening. Our results provided strong evidence for a positive, linear relationship between circadian preference and marathon completion time, whereby increasing eveningness associated with worse marathon completion time. Additionally, there was some statistical support was for a positive, linear relationship between sleep inertia severity and marathon completion time. A statistically significant interaction between circadian preference and sleep inertia on marathon completion time was also observed. However, the direction of the interaction was surprising and somewhat paradoxical. Ultimately, this study adds to the growing body of literature aimed at identifying factors that influence performance in marathons and other endurance sports, broadly. Multiple areas for future research emerged from our results, including evaluation of chronotherapy and sleep inertia countermeasures as potential strategies to enhance marathon performance.

## AUTHOR CONTRIBUTIONS

**Matthew K. P. Gratton:** Writing – original draft; conceptualization; visualization. **Jonathan Charest:** Conceptualization; writing – review and editing; data curation. **James Lickel:** Writing – review and editing. **Amy M. Bender:** Writing – review and editing; data curation. **Penny Werthner:** Writing – review and editing. **Charles R. Pedlar:** Writing – review and editing. **Courtney Kipps:** Writing – review and editing. **Doug Lawson:** Writing – review and editing. **Charles H. Samuels:** Writing – review and editing; supervision. **Jesse Cook:** Writing – original draft; conceptualization; visualization; methodology; formal analysis.

## FUNDING INFORMATION

Jonathan Charest and Amy Bender were supported by funding from MITACS (Accelerated Proposal—IT23469), with the partner being Own the Podium and The Centre for Sleep and Human Performance. This funding supported the collection and curation of the dataset. No other specific grant from any funding agency in the public, commercial or not‐for‐profit sector supported this research.

## CONFLICT OF INTEREST STATEMENT

Jesse Cook serves as a consultant to Somni©, and previously served as a consultant to Cerno Health© and Bodymatter, Inc., with these financially compensated affiliations unrelated to the current study. Amy M. Bender serves as a consultant for FIFA, Gatorade, NBA, NHL, and is on the scientific advisory board for Gainful Nutrition, with these financially compensated affiliations unrelated to the current study. Charles R. Pedlar is a consultant with Orreco, with this affiliation unrelated to the current study. All other authors declared no conflicts of interest to disclose.

## Supporting information


**FIGURE S1.** Results from the interaction analyses between circadian preference and sleep inertia on marathon completion time (minutes). This figure presents the relationship between circadian preference and marathon completion time within each level of sleep inertia. The four levels of circadian preference are plotted on the *x*‐axis of each figure pane, including Definitely a morning type (DM), More a morning type than evening type (M > E), More an evening type than a morning type (E > M), and Definitely an evening type (DE). The four levels of sleep inertia include: Very alert; Fairly alert; Slightly alert; and Not at all alert. Mean marathon completion time is presented for the level of circadian preference within the level of sleep inertia.

**FIGURE S2.** Results from the interaction analyses between circadian preference and sleep inertia on marathon completion time (minutes). This figure presents the relationship between sleep inertia and marathon completion time within each level of circadian preference. The four levels of sleep inertia are plotted on the *x*‐axis of each figure pane, including: Very alert (VA); Fairly alert (FA); Slightly alert (SA); and Not at all alert (NAAA). Mean marathon completion time is presented for the level of sleep inertia within the level of circadian preference. The four levels of circadian preference include, including Definitely a morning type (Definitely morning), More a morning type than evening type (Morning > Evening), More an evening type than a morning type (Evening > Morning), and Definitely an evening type (Definitely evening). Mean marathon completion time is presented for the level of sleep inertia within the level of circadian preference.

## Data Availability

The data that support the findings of this study are not openly available due to reasons of sensitivity, and are available from the corresponding author upon reasonable request.

## References

[jsr14375-bib-0001] Ayala, V. , Martínez‐Bebia, M. , Latorre, J. A. , Gimenez‐Blasi, N. , Jimenez‐Casquet, M. J. , Conde‐Pipo, J. , Bach‐Faig, A. , & Mariscal‐Arcas, M. (2021). Influence of circadian rhythms on sports performance. Chronobiology International, 38(11), 1522–1536. 10.1080/07420528.2021.1933003 34060402

[jsr14375-bib-0002] Bender, A. M. , Lawson, D. , Werthner, P. , & Samuels, C. H. (2018). The clinical validation of the athlete sleep screening questionnaire: An instrument to identify athletes that need further sleep assessment. Sports Med Open, 4(1), 23. 10.1186/s40798-018-0140-5 29869021 PMC5986689

[jsr14375-bib-0003] Besson, T. , Macchi, R. , Rossi, J. , Morio, C. Y. M. , Kunimasa, Y. , Nicol, C. , Vercruyssen, F. , & Millet, G. Y. (2022). Sex differences in endurance running. Sports Medicine, 52(6), 1235–1257. 10.1007/s40279-022-01651-w 35122632

[jsr14375-bib-0004] Burke, L. M. , Jeukendrup, A. E. , Jones, A. M. , & Mooses, M. (2019). Contemporary nutrition strategies to optimize performance in distance runners and race walkers. International Journal of Sport Nutrition and Exercise Metabolism, 29(2), 117–129. 10.1123/ijsnem.2019-0004 30747558

[jsr14375-bib-0005] Carrasco‐Poyatos, M. , González‐Quílez, A. , Altini, M. , & Granero‐Gallegos, A. (2022). Heart rate variability‐guided training in professional runners: Effects on performance and vagal modulation. Physiology & Behavior, 244, 113654. 10.1016/j.physbeh.2021.113654 34813821

[jsr14375-bib-0006] Cook, J. D. , Gratton, M. K. P. , Bender, A. M. , Werthner, P. , Lawson, D. , Pedlar, R. P. , Kipps, C. , Bastien, C. H. , Samuels, C. H. , & Charest, J. (2023). Sleep health, individual characteristics, lifestyle factors, and Marathon completion time in Marathon runners: A retrospective investigation of the 2016 London Marathon. Brain Sciences, 13(9), 1346. 10.3390/brainsci13091346 37759947 PMC10527296

[jsr14375-bib-0007] Costa, R. J. S. , Knechtle, B. , Tarnopolsky, M. , & Hoffman, M. D. (2019). Nutrition for ultramarathon running: Trail, track, and road. International Journal of Sport Nutrition and Exercise Metabolism, 29(2), 130–140. 10.1123/ijsnem.2018-0255 30943823

[jsr14375-bib-0008] Dibner, C. , Schibler, U. , & Albrecht, U. (2010). The mammalian circadian timing system: Organization and coordination of central and peripheral clocks. Annual Review of Physiology, 72, 517–549. 10.1146/annurev-physiol-021909-135821 20148687

[jsr14375-bib-0009] Evangelista, E. , Rassu, A. L. , Lopez, R. , Biagioli, N. , Chenini, S. , Barateau, L. , Jaussent, I. , & Dauvilliers, Y. (2022). Sleep inertia measurement with the psychomotor vigilance task in idiopathic hypersomnia. Sleep, 45(1), zsab220. 10.1093/sleep/zsab220 34436617

[jsr14375-bib-0010] Facer‐Childs, E. , & Brandstaetter, R. (2015). The impact of circadian phenotype and time since awakening on diurnal performance in athletes. Current Biology, 25(4), 518–522. 10.1016/j.cub.2014.12.036 25639241

[jsr14375-bib-0011] Furber, M. , Pyle, S. , Roberts, M. , & Roberts, J. (2021). Comparing acute, high dietary protein and carbohydrate intake on transcriptional biomarkers, fuel utilisation and exercise performance in trained male runners. Nutrients, 13(12), 4391. 10.3390/nu13124391 34959943 PMC8706924

[jsr14375-bib-0012] Haugen, T. , Sandbakk, Ø. , Seiler, S. , & Tønnessen, E. (2022). The training characteristics of world‐class distance runners: An integration of scientific literature and results‐proven practice. Sports Med Open, 8(1), 46. 10.1186/s40798-022-00438-7 35362850 PMC8975965

[jsr14375-bib-0013] Hilditch, C. J. , Dorrian, J. , & Banks, S. (2016). Time to wake up: Reactive countermeasures to sleep inertia. Industrial Health, 54(6), 528–541. 10.2486/indhealth.2015-0236 27193071 PMC5136610

[jsr14375-bib-0014] Hilditch, C. J. , & McHill, A. W. (2019). Sleep inertia: Current insights. Nature and Science of Sleep, 11, 155–165. 10.2147/NSS.S188911 PMC6710480 31692489

[jsr14375-bib-0015] Hilditch, C. J. , Pradhan, S. , Costedoat, G. , Bathurst, N. G. , Glaros, Z. , Gregory, K. B. , Shattuck, N. L. , & Flynn‐Evans, E. E. (2023). Sex differences in perceptions of sleep inertia following nighttime awakenings. SLEEP Advances, 4(1), zpac043. 10.1093/sleepadvances/zpac043 37193286 PMC10108636

[jsr14375-bib-0016] Hilditch, C. J. , Pradhan, S. , Costedoat, G. , Bathurst, N. G. , Glaros, Z. , Gregory, K. B. , Shattuck, N. L. , & Flynn‐Evans, E. E. (2023). An at‐home evaluation of a light intervention to mitigate sleep inertia symptoms. Sleep Health, 10, S121–S129. 10.1016/j.sleh.2023.07.015 37679265

[jsr14375-bib-0017] Hilditch, C. J. , Wong, L. R. , Bathurst, N. G. , Feick, N. H. , Pradhan, S. , Santamaria, A. , Shattuck, N. L. , & Flynn‐Evans, E. E. (2022). Rise and shine: The use of polychromatic short‐wavelength‐enriched light to mitigate sleep inertia at night following awakening from slow‐wave sleep. Journal of Sleep Research, 31(5), e13558. 10.1111/jsr.13558 35102669 PMC9787581

[jsr14375-bib-0018] Kanady, J. C. , & Harvey, A. G. (2015). Development and validation of the sleep inertia questionnaire (SIQ) and assessment of sleep inertia in analogue and clinical depression. Cognit Ther Res, 39(5), 601–612. 10.1007/s10608-015-9686-4 PMC4593308 26451063

[jsr14375-bib-0019] Lopes, T. R. , Pereira, H. M. , Bittencourt, L. R. A. , & Silva, B. M. (2023). How much does sleep deprivation impair endurance performance? A systematic review and meta‐analysis. European Journal of Sport Science, 23(7), 1279–1292. 10.1080/17461391.2022.2155583 36472094

[jsr14375-bib-0020] Lundholm, K. R. , Honn, K. A. , Skeiky, L. , Muck, R. A. , & Van Dongen, H. P. A. (2021). Trait Interindividual differences in the magnitude of subjective sleepiness from sleep inertia. Clocks Sleep, 3(2), 298–311. 10.3390/clockssleep3020019 34204864 PMC8293243

[jsr14375-bib-0021] Marsden, R. (2016). London Marathon 2016: route, course map, times, road closures and event details. Retrieved 9/2/2024 from https://bleacherreport.com/articles/2634560-london-marathon-2016-route-course-map-times-road-closures-and-event-details.

[jsr14375-bib-0022] Neves, A. R. , Albuquerque, T. , Quintela, T. , & Costa, D. (2022). Circadian rhythm and disease: Relationship, new insights, and future perspectives. Journal of Cellular Physiology, 237(8), 3239–3256. 10.1002/jcp.30815 35696609

[jsr14375-bib-0023] Nikolaidis, P. T. , Alvero‐Cruz, J. R. , Villiger, E. , Rosemann, T. , & Knechtle, B. (2019). The age‐related performance decline in Marathon running: The paradigm of the Berlin Marathon. International Journal of Environmental Research and Public Health, 16(11), 2022. 10.3390/ijerph16112022 31174325 PMC6603944

[jsr14375-bib-0024] Nobari, H. , Azarian, S. , Saedmocheshi, S. , Valdés‐Badilla, P. , & García Calvo, T. (2023). Narrative review: The role of circadian rhythm on sports performance, hormonal regulation, immune system function, and injury prevention in athletes. Heliyon, 9(9), e19636. 10.1016/j.heliyon.2023.e19636 37809566 PMC10558889

[jsr14375-bib-0025] Prieto‐González, P. , & Sedlacek, J. (2022). Effects of running‐specific strength training, endurance training, and concurrent training on recreational endurance Athletes' performance and selected anthropometric parameters. International Journal of Environmental Research and Public Health, 19(17), 10773. 10.3390/ijerph191710773 36078489 PMC9518107

[jsr14375-bib-0026] Putilov, A. A. (2017). Owls, larks, swifts, woodcocks and they are not alone: A historical review of methodology for multidimensional self‐assessment of individual differences in sleep‐wake pattern. Chronobiology International, 34(3), 426–437. 10.1080/07420528.2017.1278704 28128994

[jsr14375-bib-0027] Reusser, M. , Sousa, C. V. , Villiger, E. , Alvero Cruz, J. R. , Hill, L. , Rosemann, T. , Nikolaidis, P. T. , & Knechtle, B. (2021). Increased participation and decreased performance in recreational master athletes in ‘Berlin Marathon’ 1974–2019. Frontiers in Physiology, 12, 631237. 10.3389/fphys.2021.631237 34262467 PMC8273432

[jsr14375-bib-0028] Richardson, J. T. E. (2011). Eta squared and partial eta squared as measures of effect size in educational research. Educational Research Review, 6(2), 135–147. 10.1016/j.edurev.2010.12.001

[jsr14375-bib-0029] Samuels, C. , James, L. , Lawson, D. , & Meeuwisse, W. (2016). The athlete sleep screening questionnaire: A new tool for assessing and managing sleep in elite athletes. British Journal of Sports Medicine, 50(7), 418–422. 10.1136/bjsports-2014-094332 26002952

[jsr14375-bib-0030] Smyth, B. , & Muniz‐Pumares, D. (2020). Calculation of critical speed from raw training data in recreational Marathon runners. Medicine and Science in Sports and Exercise, 52(12), 2637–2645. 10.1249/MSS.0000000000002412 32472926 PMC7664951

[jsr14375-bib-0031] TCS London Marathon . (2023). Good for age entry. Retrieved 9/9/2023 from https://www.tcslondonmarathon.com/enter/how-to-enter/good-for-age-entry.

[jsr14375-bib-0032] TCS London Marathon . (2024). Explore the TCS London Marathon course. Retrieved 9/2/2024 from https://www.tcslondonmarathon.com/the-event/the-course.

[jsr14375-bib-0033] Trotti, L. M. (2017). Waking up is the hardest thing I do all day: Sleep inertia and sleep drunkenness. Sleep Medicine Reviews, 35, 76–84. 10.1016/j.smrv.2016.08.005 27692973 PMC5337178

[jsr14375-bib-0034] Williams, V. S. L. , Jones, L. V. , & Tukey, J. W. (1999). Controlling error in multiple comparisons, with examples from state‐to‐state differences in educational achievement. Journal of Educational and Behavioral Statistics, 24(1), 42–69. 10.3102/10769986024001042

