# Supplementary figures and images for: Influence of circadian preference, sleep inertia and their interaction on marathon completion time: A retrospective, cross‐sectional investigation of a large mass‐participation city marathon
Source: J Sleep Res. 2024 Oct 19;34(3):e14375. doi: 10.1111/jsr.14375 (PMC12069743; doi:10.1111/jsr.14375)

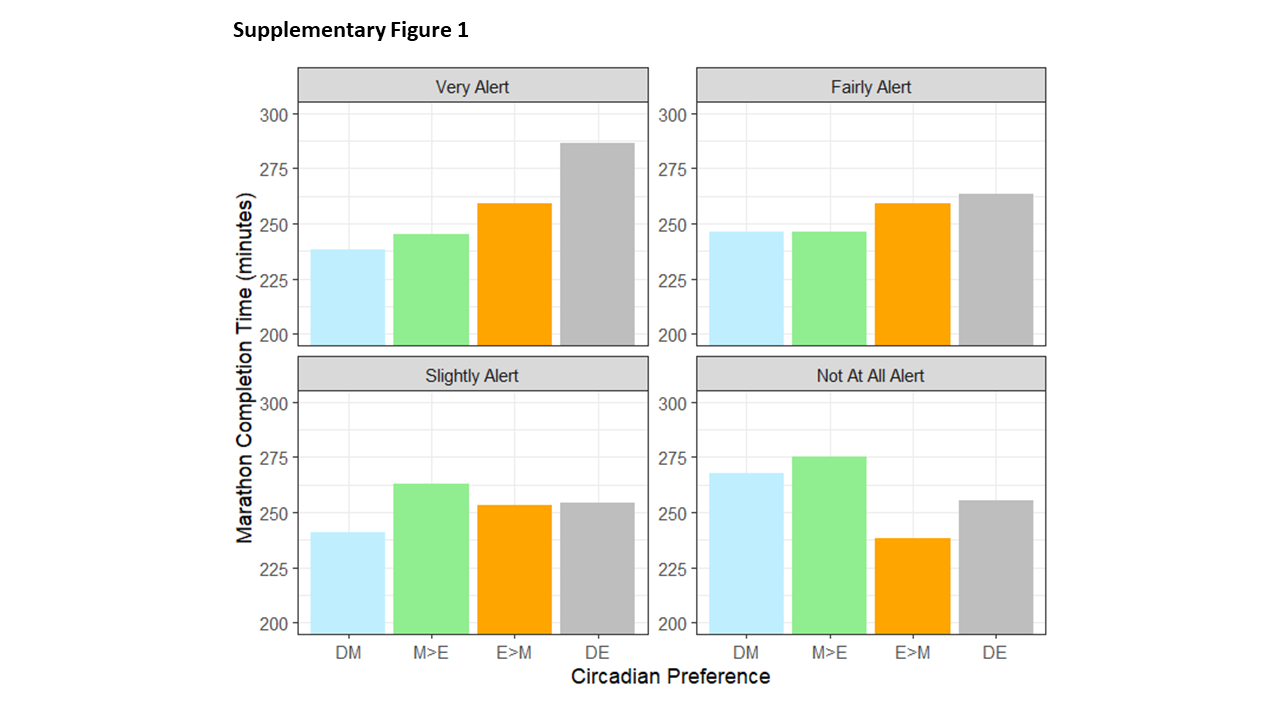

Supplement: Supplementary file 1 — FIGURE S1. Results from the interaction analyses between circadian preference and sleep inertia on marathon completion time (minutes). This figure presents the relationship between circadian preference and marathon completion time within each level of sleep inertia. The four levels of circadian preference are plotted on the x‐axis of each figure pane, including Definitely a morning type (DM), More a morning type than evening type (M > E), More an evening type than a morning type (E > M), and Definitely an evening type (DE). The four levels of sleep inertia include: Very alert; Fairly alert; Slightly alert; and Not at all alert. Mean marathon completion time is presented for the level of circadian preference within the level of sleep inertia. [file JSR-34-e14375-s001.tif]

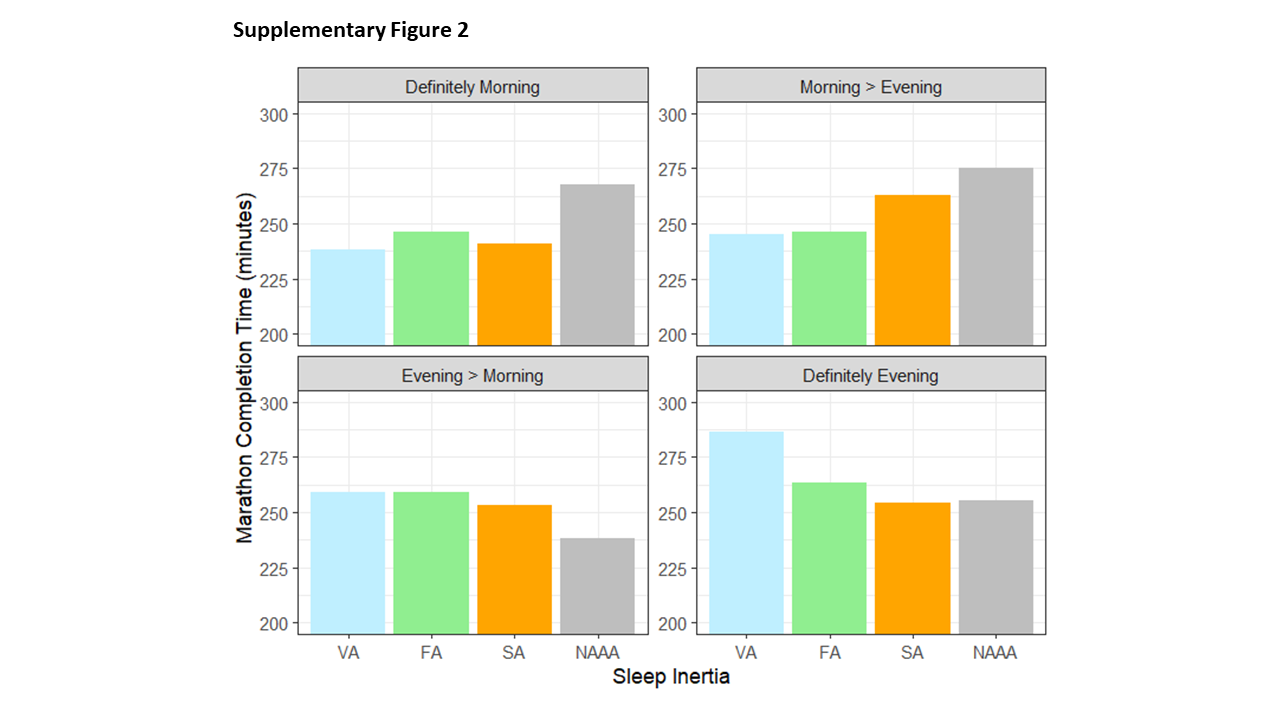

Supplement: Supplementary file 2 — FIGURE S2. Results from the interaction analyses between circadian preference and sleep inertia on marathon completion time (minutes). This figure presents the relationship between sleep inertia and marathon completion time within each level of circadian preference. The four levels of sleep inertia are plotted on the x‐axis of each figure pane, including: Very alert (VA); Fairly alert (FA); Slightly alert (SA); and Not at all alert (NAAA). Mean marathon completion time is presented for the level of sleep inertia within the level of circadian preference. The four levels of circadian preference include, including Definitely a morning type (Definitely morning), More a morning type than evening type (Morning > Evening), More an evening type than a morning type (Evening > Morning), and Definitely an evening type (Definitely evening). Mean marathon completion time is presented for the level of sleep inertia within the level of circadian preference. [file JSR-34-e14375-s002.tif]
